# How ecological feedbacks between human population and land cover influence sustainability

**DOI:** 10.1371/journal.pcbi.1006389

**Published:** 2018-08-17

**Authors:** Kirsten Henderson, Michel Loreau

**Affiliations:** Centre for Biodiversity, Theory, and Modelling, Theoretical and Experimental Ecology Station, CNRS, Moulis, France; University of Chicago, UNITED STATES

## Abstract

It is estimated that the Earth’s biocapacity is unable to meet current demands, which begs the question: is a sustainable future possible for both humans and the environment? The UN projects a human population of approximately 11 billion by the end of the 21st century; requiring additional agricultural land, greater demands for natural resources, and technological advancements. We model human population over the next century, emphasizing feedbacks between natural and agricultural resource availability and human demography. We argue that an intensive agriculture approach to feeding the growing population is ill-conceived, without considering biodiversity and ecosystem services (e.g., nutrient cycling, pollination, water purification, pest control). The productivity of agricultural land and human population dynamics are dependent on the area of natural land—generally, tipping at 5 billion ha of natural land (approximately 40% of the Earth’s terrestrial area). Furthermore, our model shows that an imprudent proactive approach (i.e., focusing on agriculture and ignoring ecosystem services) limits the success of reactive measures (i.e., restoration) in the future, while the inability to react to changes and recover natural systems leads to human population decline.

## Author summary

There exist human population projections and models of agricultural expansion or land-use change patterns. However, the ability to combine feedbacks and concepts of population growth and land-use change into a simple dynamical model is a unique feature that allows us to firstly generate realistic predictions using resource use and demographic data. Second, evaluate alternative scenarios, predict collapse and highlight opportunities for sustainability.

## Introduction

The current human population size seems almost inconceivable when compared to the history of humanity—about 2000 times greater than 10 000 BCE. The population grew at a relatively low and steady rate between 10 000 BCE and 1700 (0.04% annually), followed by a drastic increase after 1760, with population growth consistently above 0.4% and peaking at 2.1% (about 50 times greater than 1700) in the 1960s [1]. No single driver can explain this phenomenon, rather it is a combination of many factors, including human demographics, cultural values, ecosystem services, economic values and institutional practices. Notably, the increase in consumption over the last 2000 years has grown in tandem with human population, especially after 1750 [2], asserting that demographics, economics, inequality and ecosystems are intricately linked [3]. Furthermore, Ceballos and colleagues [4] suggest that these factors, namely, overpopulation, overconsumption, and economic inequality mar the idea that growth can continue on a finite planet. The assumption that population and ecosystem dynamics are autonomous is a belief that has triggered past societal collapses and could potentially lead to unsustainable trajectories in the future.

There is a general consensus that past local and regional civilization collapse resulted from deteriorated land productivity followed by the inability to feed individuals. In most cases of civilization collapse, the land became inhospitable to the once established society, whether from volcanic eruption (North Mesopotamian Civilization [5]), soil degradation (Norse), drought (Mayan) or resource exploitation (Easter Island) [6]. No matter the underlying cause of environmental change, the failure to recognize over-exploitation of resources in a changing environment ultimately led to the demise of these civilizations; where the civilization continued along the normal course, until one day the bottom fell out [7, 8].

The inability to change perceptions and habits is thought to be responsible for the collapse of many civilizations. These cultural failures are linked to social and economic organization in societies that limit opportunities through myopic views [6]. For example, the Norse preferred to continue farming on degraded land rather than transition to a fish-based diet, and the Easter Islanders continued to make ahu and maoi figures despite dwindling forests. Alternatively, the ability to change behaviours and practices can reduce the impact or immunize the society against collapse. The successful transition from hunter-gatherer to plant cultivation and animal rearing in new locales prevented the Natufians of Southwest Asia from population collapse.

Today, the focus is placed on agriculture to feed the growing population. However, often neglected are the ecosystem services that supply agricultural systems: soil fertility, nutrients, water provision and purification, pollination, genetic biodiversity, atmosphere regulation, pest control [9, 10]. In turn, many agriculture systems act as a siphon, taking resources from natural land and giving little to nothing in return. In the short-term, there are evident anthropogenic benefits to intensive agriculture. The surge in food production over the last 60 years can be attributed to the Green Revolution, but these practices also contribute significantly to environmental damage [11].

There are multiple feedbacks between the human, natural and agricultural systems. Motesharrei and colleagues [3] argue that the feedbacks between the Earth and human systems must be incorporated in the models to avoid underestimating environmental challenges that humanity faces, and to avoid missing important non-trivial dynamics of the coupled system. They suggest that because of these feedbacks, changes in the environment can affect human health, reduce economic productivity and decrease agricultural yields. For example, increased consumption of water in densely populated cities can result in lower agricultural yields, as groundwater diminishes and freshwater quality declines. This over-exploitation of ecosystem services raises concerns about the long term sustainability of intensive agriculture.

There are many parallels in today’s society with earlier civilizations, the major difference being that we are a global society, which may potentially have much greater implications if a collapse were to occur. The Earth is not limitless. There exist planetary bounds and as such it is axiomatic that demands should not surpass biocapacity and available resources; that population levels cannot continue to grow with the same use of resources and agriculture; that the rapid depletion of non-renewable resources, without seeking renewable alternatives, is unsustainable. Individuals throughout history, including today, share in the ignorance about these concepts, believing in a cornucopian existence. Previous examples of collapse occurred unbeknownst to the individuals. We do, however, have a distinct advantage over societies in the past because we can anticipate the future [12]. Our power of foresight is not perfect, but models allow us to highlight potential shortcomings and feedbacks in social, ecological and economic practices [2, 3].

The purpose of this work is to highlight the driving factors in human and ecosystem sustainability, by first creating a simple dynamical model, with bidirectional feedbacks between the human population and land cover, that generates projections into the next century, and then analysing potential opportunities for a sustainable human population with adequate resources, while maintaining biodiversity and ecosystem services. Besides the obvious ways of reducing human impact on the ecosystems and attempting to achieve sustainability, which include reducing demand, developing technological solutions and reducing the human population [13], we seek alternative strategies for maintaining population levels. We compare proactive shifts in human behaviour and consumption patterns, with reactive measures that restore land once degraded or increase land area to meet demands (i.e., land conversion or restoration).

## Methods

### Model

**Fig 1:**
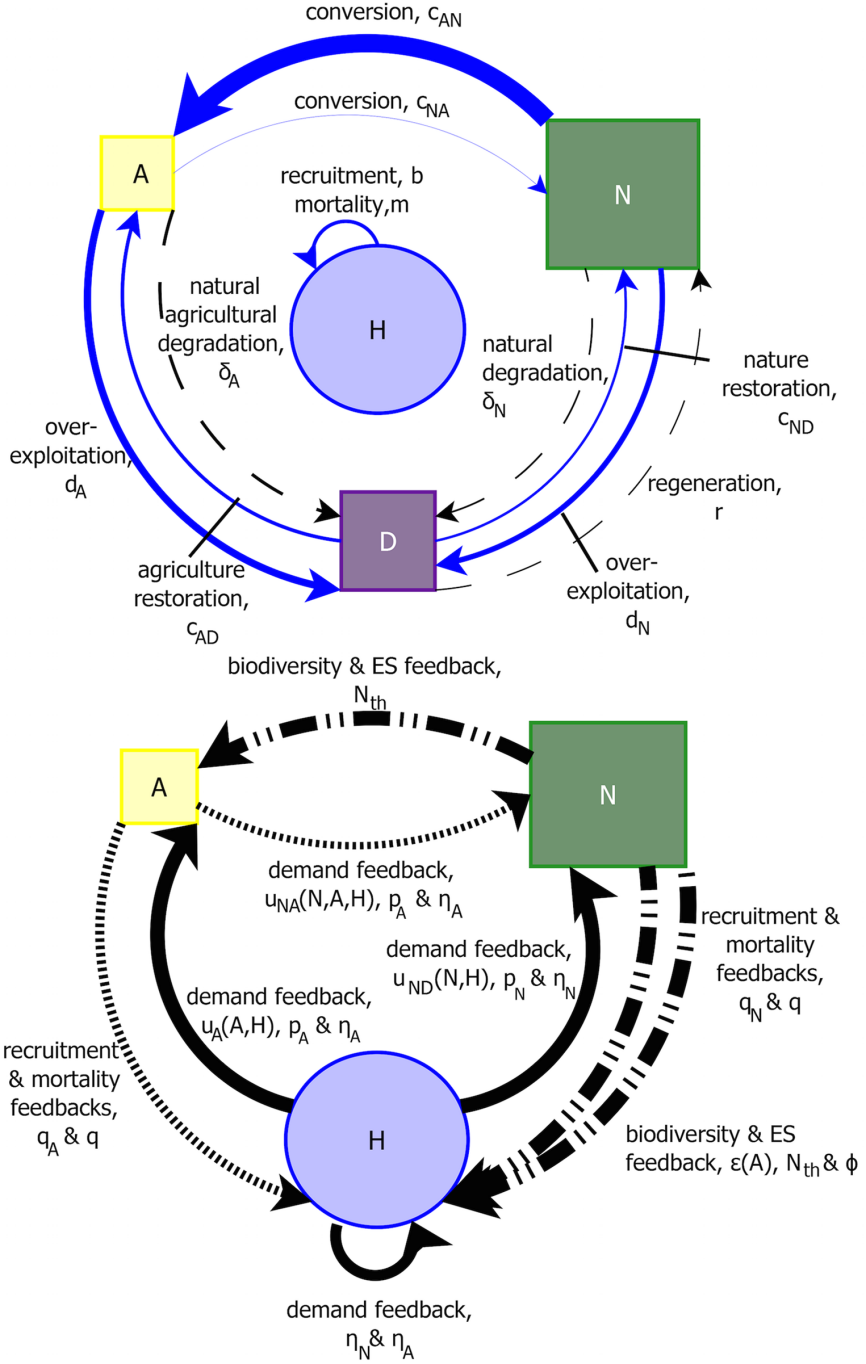
Conceptual diagram of the model. We consider a four-variable system of natural/‘semi-natural’ land (*N*), intensive agriculture (*A*), unproductive/degraded land (*D*) and human population (*H*). The left panel depicts forces on the system, either anthropogenic (in blue) or natural (in black). The right panel represents feedbacks in the model: solid lines are human-driven feedbacks, dashed lines are ecosystem service (ES) feedbacks, and dotted lines are food resource feedbacks. In the right panel, the model has been simplified to a two land variable system, where *N* + *A* + *D* = *L*. *L* refers to the total terrestrial land area on Earth. The width of the arrows represent the rate or impact of the parameter. The parameter values and ranges are given in Table A in S1 Text.

The model is conceptualized in Fig 1, depicting three land variables: productive natural/‘semi-natural’ land (*N*), intensive agriculture (*A*) and unproductive/degraded land (*D*); in addition to the human population (*H*). The majority of forces on the system are imposed by the human population (Fig 1a), while many of the feedbacks are the result of ecological constraints (Fig 1b). The model compares different pathways and approaches to sustainability, through: demography, resource use, land conversion and natural forces/feedbacks. These categories are further described in terms of proactive and reactive measures, given in greater detail below.

This coupled human-environment model describes the feedbacks between human demography, resource use and land area changes. Natural land is not easily defined, as much of the Earth’s ice-free surface is directly impacted (estimated to be 75-83%) by human behaviour [14]. We consider productive natural/‘semi-natural’ land (*N*) in our model to be land that provides ecosystem services (ES), while maintaining biodiversity: forest area (natural but not necessarily virgin), meadows and pastures (mostly uncultivated). Permanent crops, monocultures, and arable land area constitute *A*. Degraded or unproductive land is an umbrella term describing any land that has been degraded by agriculture and forestry, human infrastructure and other land that includes desert, tundra, land unsuitable for agriculture, and high mountains.

Natural land cover is influenced by both natural and human forces. In addition, conservation efforts apply scarcity-driven, supply and demand concepts, discussed in further detail below. The change in natural land (*N*) is given by:

$$\begin{array}{c} \begin{array}{c} \dot{N}=rD+c_{{}_{NA}}u_{{}_{NA}}\left(N,A,H\right)HA+c_{{}_{ND}}u_{{}_{ND}}\left(N,H\right)HD-\\ c_{{}_{AN}}u_{A}\left(A,H\right)NH-d_{N}HN-\delta_{N}N, \end{array} \end{array}$$ (1)

where *r* is the natural regeneration rate from degraded/unproductive land (*D*) to natural land (*N*). Natural disturbance or degradation (i.e., fire, wind, drought) is represented by *δ*_*N*_. In the absence of human influence the system converges to a combination of *N* and *D*, depending on the rate of regeneration and degradation. As human population (*H*) increases, there is an increase in land change [11, 15]. Without replenishing the natural area or by depleting the land too quickly, such anthropogenic practices can lead to lost biocapacity. *d*_*N*_, human-driven degradation, is the proportion of *N* that is harvested or consumed each year and is not returned to its original state, as a result of over-exploitation and degradation. Conversion to *N* occurs through conservation/restoration efforts (*c_NA_*, *c_ND_*), which are assumed to be influenced by scarcity [16]. Scarcity is reflected by perception valuation *u_ND_*(*N*, *H*), described below. *c_NA_* is the maximum proportion of agricultural land (*A*) converted to natural land (*N*). This term is small, as agriculture is unlikely to be converted to natural land based on perceptions of land value *u_NA_*(*N*, *A*, *H*). *c_ND_* is the maximum proportion of *D* converted to *N*. As population grows or individuals increase their consumption patterns, the demand for *A* increases, such that *c_AN_* is the maximum conversion of *N* to *A*. Conversion to *A* also involves perception [17], *u_A_*(*A*, *H*), discussed below.

Changes in agricultural land (*A*) are driven primarily by human factors. The change in *A* is given by:

$$\begin{array}{c} \begin{array}{c} \dot{A}=c_{{}_{AN}}u_{A}\left(A,H\right)NH+c_{{}_{AD}}u_{A}\left(N,H\right)HD-\\ c_{{}_{NA}}u_{{}_{NA}}\left(N,A,H\right)HA-d_{A}\exp\left(-N/N_{th}\right)HA-\delta_{A}A. \end{array} \end{array}$$ (2)

The conversion of *D* to *A* is less common than natural land, particularly forest area [18]; nonetheless, *c_AD_* describes the maximum proportion of *D* to *A*. *d*_*A*_ is the rate of degradation caused by human consumption and over-exploitation of *A*. The rate of anthropogenic degradation of *A* (*d*_*A*_) can be slowed by the availability of ES and biodiversity [19], described by the term, exp(−*N*/*N*_*th*_). There exist positive feedbacks between agriculture conversion and forest cover decline. In many regions of the world forest is rapidly converted into agriculture, to such an extent that soil quality is degraded, which reduces crop yield and ultimately results in greater conversion [20].

*N*_*th*_ is the threshold of *N* needed to maintain biodiversity and ES supplied to agricultural systems. High-species diversity in ecosystems often promote greater ecosystem services [21], in some cases increasing crop yields by 60% [22]. Maintaining species requires at least 25% of the natural habitat area [23]. Furthermore, many studies show that agricultural systems with 30% natural land area result in improved pollination [24]. Pollination is just one of the many benefits agriculture receives from nature, in addition to nutrient cycling, pest control, increased yield and water filtration [25, 10]. *N*_*th*_ is estimated to be approximately 40% of the terrestrial surface area, based on optimal pollination studies, habitat area studies and the dependence of agriculture on regulating and supporting services. As there is no consensus for such a threshold we provide alternative thresholds and give greater details in S1 Text.

The human population acts as a multiplier of activities, such as consumption, environmental damage, and land conversion. Without humans, *A* would disappear at a rate of *δ*_*A*_—the rate at which *A* naturally degrades, as a result of fire, erosion, drought, nutrient cycling.

The perceived value of land ultimately determines how much land is converted into *A* or *N* [26, 27]. For example, today’s society offers more incentives for agricultural purposes and there is a general opinion is that there is a greater need for agricultural land [19]. The following equations describe the collective perception of land value:

$$\begin{array}{c} u_{{}_{NA}}\left(N,A,H\right)=\max(0,\frac{\eta_{N}H-\left(1-p_{N}\right)N-\eta_{A}H+\left(1-p_{A}\right)A}{L}), \end{array}$$ (3a)

$$\begin{array}{c} u_{{}_{ND}}\left(N,H\right)=\max(0,\frac{\eta_{N}H-\left(1-p_{N}\right)N}{L}), \end{array}$$ (3b)

$$\begin{array}{c} u_{A}\left(A,H\right)=\max(0,\frac{\eta_{A}H-\left(1-p_{A}\right)A}{L}). \end{array}$$ (3c)

In order to convert *A* to *N* the demand for nature (*η*_*N*_) must be greater than the demand for agriculture (*η*_*A*_), based on the assumption that agricultural land demands must be met before the population considers converting *A* to *N*. Deterministic decision-making, including supply and demand principles, is reflected by the above equations. Conversion of *A* and *N* increases as demand increases; however, humans are not always rational [28]. Hence, *p*_*N*_ and *p*_*A*_ represent a perceived value of *N* and *A*, respectively. As *p* approaches 0, the value of land is based on deterministic supply and demand requirements, rather than perceived value. When *p* approaches 1 the decisions are entirely based on perceived value, rather than supply and demand rationalization.

Degraded/unproductive land (*D*) changes as a result of human actions and natural disturbance to *N* and *A*, such that $\dot{N}+\dot{A}+\dot{D}=0$. The equation for changes in *D* are given by

$$\begin{array}{c} \begin{array}{c} \dot{D}=d_{N}HN+\delta_{N}N+d_{A}\exp\left(-N/N_{th}\right)HA+\delta_{A}A\\ -c_{ND}u_{{}_{ND}}\left(N,H\right)HD-c_{{}_{AD}}u_{A}\left(A,H\right)HD. \end{array} \end{array}$$ (4)

The main driver in natural/‘semi-natural’ land (*N*) and agricultural land (*A*) changes is human population. In turn, land dynamics feedback to influence human population demography. The change in human population size (*H*) is given by:

$$\begin{array}{c} \dot{H}=be^{-\beta \eta_{N}}\left(1-\exp\left(-q_{N}N-q_{A}A\right)\right)H-m\exp\left(q(\eta_{N}H-E_{N}N+\eta_{A}H-\epsilon_{A}\left(N\right)A)\right)H, \end{array}$$ (5)

where *b* is the average recruitment rate. The recruitment rate refers to the birth rate minus the under-five mortality rate. Prior to the industrial revolution, human population growth seemed to follow a Malthusian model, where resources dictated growth patterns [29]. Many earlier models use this idea to describe population growth [30], although this may not be evident today, as higher birth rates occur in regions with minimal food. However, the combined term, recruitment, can still be explained by resource availability. Under-five mortality rates are strongly correlated with nutrition and rural living [31, 32]. Regions with more land to cultivate and less technology require greater human capital, which increases the birth rate. Additionally, higher infant mortality rates indirectly lead to higher birth rates, as a result of discrepancies between perceived number of offspring and actual number of offspring [33].

It is assumed that the current recruitment rate cannot be sustained without resources from *N* and *A* [34, 35], therefore *q*_*N*_ and *q*_*A*_ represent the labour coefficients for natural and agricultural resources. This model is applied to a range of potential scenarios, beyond the current trends and predictions, therefore the term 1 − exp((−*q*_*N*_ *N* − *q*_*A*_ *A*)) is used to reflect extreme scenarios, where *N* → 0 or *A* → 0 and the recruitment rate collapses. Otherwise, the influence of *q*_*N*_ and *q*_*A*_ on recruitment rate is minimal. *β* is a coefficient which reflects the demographic transition, where the recruitment rate decreases as consumption of natural resources increases [36]. We let natural land resource demand (*η*_*N*_) be a proxy for affluence and education, which results in decreased recruitment rates [37]. *m* is the average mortality rate of individuals above the age of five. Here we assume that natural and agricultural resource availability, in addition to efficiency of use, decrease mortality [7, 31]; the influence of resources on mortality is reflected by *q*. *E*_*N*_ and *ϵ*_*A*_(*N*) describe the efficiency of use, harvest and distribution for *N* and *A*, respectively.

As is the case with anthropogenic degradation of agriculture (*d*_*A*_(*N*)), the efficiency of use of agricultural resources (*E*_*A*_) decreases with insufficient input from ES and low biodiversity. *E*_*A*_ may increase through improved distribution, reduced waste and technological improvements. However, there is a strong link between energy, food and water, which can limit yield, despite improvements in other sectors. Therefore, agricultural efficiency should also include interactions with *N* and ES [38, 25]. Efficiency of agricultural land as a function of *N* is given by:

$$\begin{array}{c} \epsilon_{A}\left(N\right)=\max\left(0,E_{A}-\phi \left(N_{th}-N\right)\right) \end{array}$$ (6)

where *ϕ* is a coefficient that determines the influence of *N*_*th*_ on agricultural benefits to humans. Parameter justification and ranges are supplied in S1 Text.

### Analysis

We are interested in the forces and feedbacks dictating population growth and land cover change. We group model parameters—proactive and reactive measures, human-driven and nature-driven change, parameters that influence human population growth or parameters that govern land cover change—to determine what best describes mechanisms of change in human populations and land use.

The term proactive describes any change in human behaviour or land-use that acts in anticipation of future issues, practices or demands (*p*_*N*_, *p*_*A*_, *d*_*N*_, *d*_*A*_); reactive measures are those that act in response to a situation, for example restoration of land once it has become scarce or increasing land area to meet needs (*c_NA_*, *c_AN_*, *c_ND_*, *c_AD_*). We consider consumption requirements (*η*_*N*_, *η*_*A*_) and technological advancements (*E*_*N*_, *E*_*A*_) to be proactive changes that alter the way the collective human population uses resources.

## Results

### Business as usual

**Fig 2:**
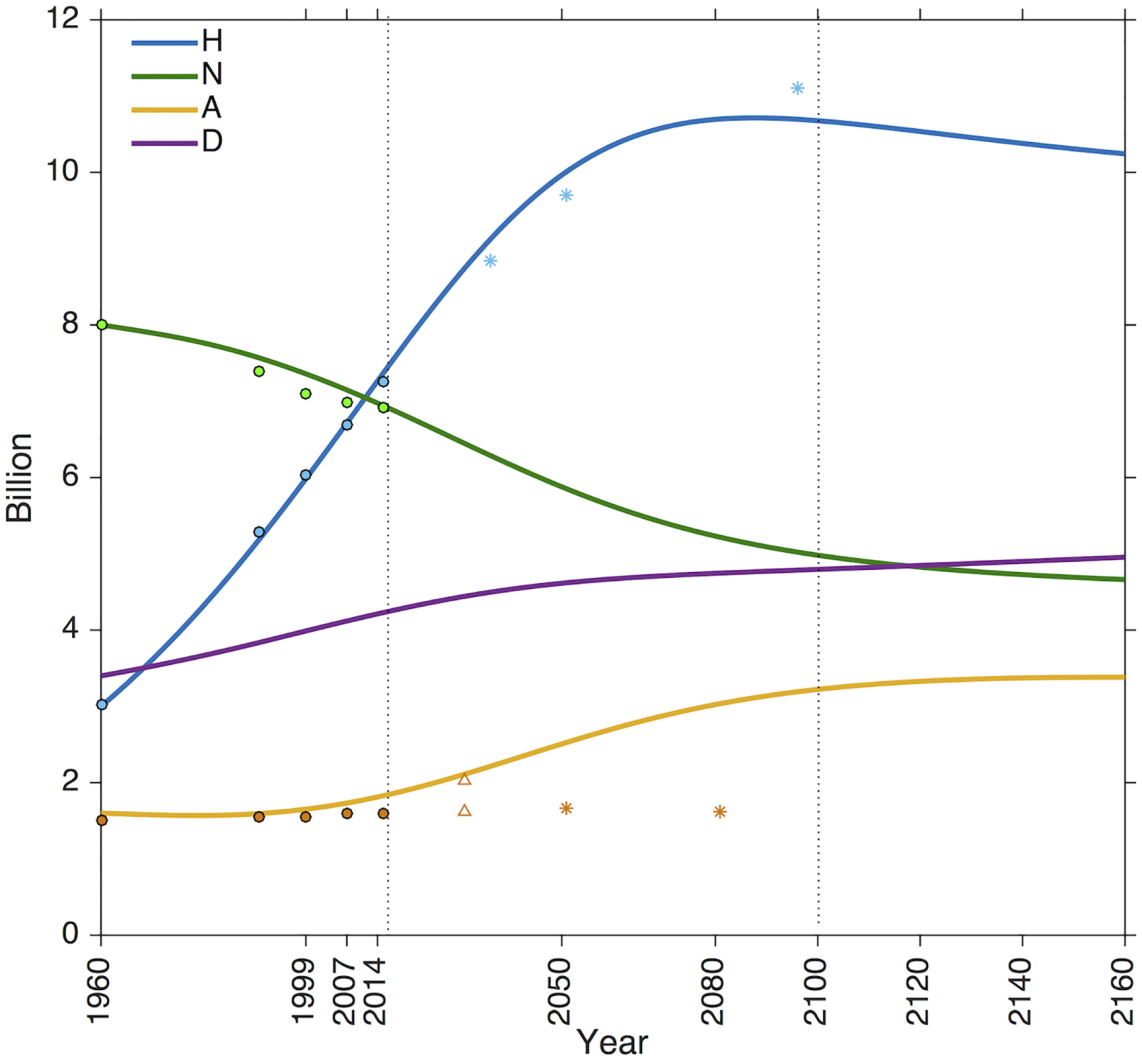
Population and land cover dynamics under a business as usual scenario. Empirical data from 1960 to 2016 was used to fit the model (filled circles). Between the two dotted lines are the projections through to the next century with other model projections for reference (asterisks/triangles). Simulations show a human population (*H*) peak at the end of the 21st century and then a decline, as demands are no longer met. Agricultural land area (*A*, ha) has stayed relatively constant over the last 50 years and will double by the next century. FAO projections [49], which assume that the majority of increased agricultural production will come from increased intensity, are represented by asterisks. Lambin and colleagues offer a high and low estimate for *A* in 2030 represented by triangles [15]. Change in *N* is primarily driven by agricultural conversion. The change in *A* is driven by the growing population (*H*). Once *N* can no longer support the high demand for *A*, as a result of over-consumption, *H* decreases. The area of unproductive and degraded land (*D*) increases with *H* as a result of degradation from over-consumption and demand for urban area. Past *H* and *A* data was obtained from the World Bank database [55]. Details on empirical estimates for variables are provided in S1 Text.

Model simulations reveal that the business as usual scenario doubles agricultural area (*A*) over the next century, while decreasing natural and ‘semi-natural’ land (*N*) by 40% and increasing degraded/unproductive land by 17% (Fig 2). The population continues to grow until 2080, reaching 10.8 billion individuals, and slowly drops off at the end of the century.

### Alternative scenarios

By looking at alternative scenarios we can gauge what is truly driving the system. Model parameters that are driven by human decisions or practices, especially those that govern resource consumption and demand, have the largest influence on peak values, equilibrium values and the range of possible dynamics (Fig B in S1 Text). Parameters that increase the productive natural and ‘semi-natural’ land area (*N*) also increase the area of agricultural land (*A*), decrease unproductive land area (*D*) and either maintain or increase human population (*H*). By contrast, model parameters that boost agriculture allow the human population to grow quickly and increase the peak population, but without sufficient *N* (and by proxy ecosystem services), the human population declines. This decline in *H* is the result of increased mortality from a lack of *N* (i.e., provisioning and regulating services). There is adequate agricultural area, but the quality of agriculture is reduced from a lack of *N* (i.e., regulating services), which negatively impacts humans. Additional details on parameter significance and model simplification is provided in S1 Text.

Parameters that govern demographics (*m*, *b*), resource use (*η*_*N*_, *η*_*A*_, *d*_*N*_, *p*_*A*_), and ES feedbacks on human population growth (*N*_*th*_, *q*) are the most strongly influential components of the model, many of which are dictated by the human population. By artificially inflating or decreasing recruitment (*b*) and mortality (*m*) rates, it can be shown that demographic parameters influence the peak population and the onset of population decline; however, changing *b* and *m* does not prevent the population from collapsing. Changing *b* and *m* merely delays (low mortality rates, mid-range recruitment rates) or accelerates (very low recruitment rates, high mortality rates) the onset of population decline, as a consequence of consumption and technology remaining constant in the model. By contrast, the natural feedback of resource availability and ES on mortality, *q*, strongly impacts whether there is a decline in the population. This coefficient *q* is unknown and is scaled to fit the model to demographic data and therefore, we cannot know for certain how great the influence of ES or food resources are on human well-being. Nonetheless, this coefficient gives an indication of the prominent influence of resource availability on human well-being, suggesting that demographic trends are sensitive to resource availability and ES, and vice versa.

Thus far the model results have alluded to the importance of maintaining natural land for ecosystem and human sustainability through parameter analyses. More specifically, natural land (*N*) degradation creates sharp decreases in human population (*H*)—much more notable than the decline in agricultural land (*A*). In all the scenarios evaluated (business as usual, changing perceptions of land value, and comparing proactive/reactive measures) and by modifying parameters (Fig B in S1 Text), it can be seen that maintaining approximately 5 billion ha of *N* globally (38% of the Earth’s terrestrial surface area) is essential to maintaining the human population size. Once *N* falls below 5B ha, the agricultural yield diminishes and the human population declines as a result of declines in agricultural yield (indirect effect of declining *N*) and decreased provisioning services from *N* (direct effect). The tipping point is susceptible to shifts, depending on resource use parameters and the rate of restoration (Fig B in S1 Text). A decrease in demand (*η*_*N*_, *η*_*A*_) delays the initiation of decline and requires less *N* to maintain *H*. Furthermore, greater restoration efforts prevent *H* decline. From model simulations, we find that maintaining sufficient *N* is the most evident way to sustain both the human population and agricultural productivity (more details are given in the alternative scenarios described below). However, once natural land is degraded beyond a critical point, the demand for resources and ecosystem services is too great, which results in a human population decline. Although most scenarios have a tipping point of approximately 5B ha, the diversity of the tipping point seen in figure B in S1 Text can also be explained by the rate of *N* decline (see details in Fig A in S1 Text).

#### Perception bias

**Fig 3:**
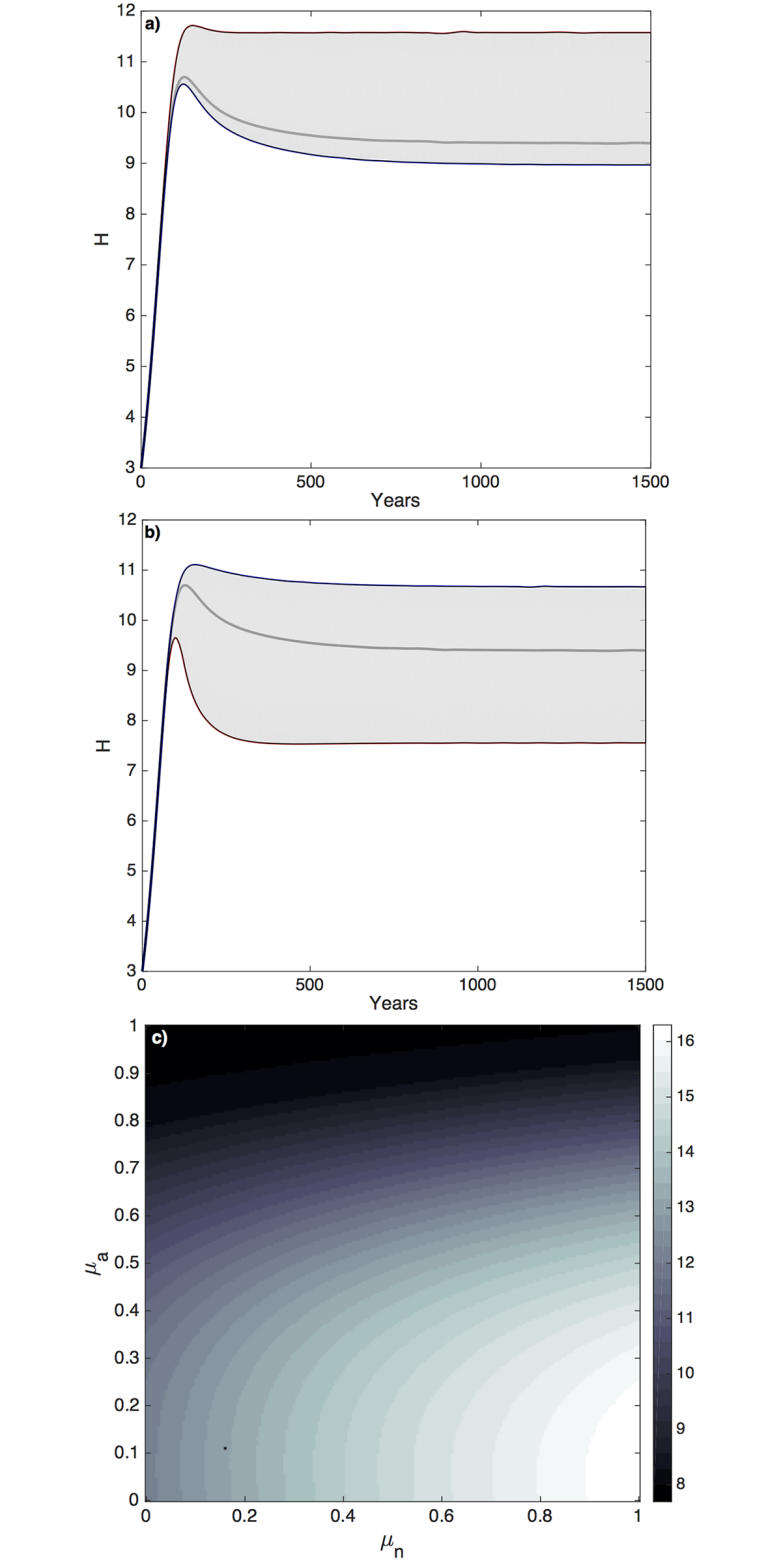
A high perceived value of agricultural land (*μ*_*a*_) leads to a counterproductive decrease in human population size (*H*). **a)** Increasing an individual’s perceived value of natural land (red line) results in a higher stable *H* and a very low perceived value of *N* (blue line) leads to decline. **b)** Contrarily, increasing the collective perceived value of agricultural land (red line) corresponds to a large population decline. The black line in a) and b) represents business as usual scenario. **c)** Parameter plane of the human population equilibrium over a range of collective perceived values of *N* (*p*_*N*_) and *A* (*p*_*A*_), where the highest sustained population levels occur when agricultural perceived value is low (*p*_*A*_ = 0), based on deterministic supply and demand, rather than perception bias.

An unanticipated result of the model relates to how the collective human population perceives the value of land or resources. A higher perceived value of agricultural land (*p*_*A*_) increases agricultural area (*A*), but without the supporting biodiversity and ecosystem services (*N*), the human population (*H*) is not sustained; ultimately, this results in a lower population peak, followed by a sharp decline (Fig 3b, red line). By contrast, a high perceived value of natural land (*p*_*N*_) increases *A* and *H*, in addition to *N*. This increase in *A* and *H* is then followed by a decline; however, there is a more gradual and smaller decline in human population, as natural land area decreases more slowly with *p*_*N*_ → 1 (Fig 3a). It can be seen from simulations (Fig 3c) that the counterintuitive negative impact of having a high perceived value of agricultural land (*p*_*A*_) outweighs the beneficial components of having a high perceived value of natural land (*p*_*N*_). Higher equilibrium populations are achieved as *p*_*N*_ → 1, without changing resource use or further developing technology.

#### Proactive and reactive measures

**Fig 4:**
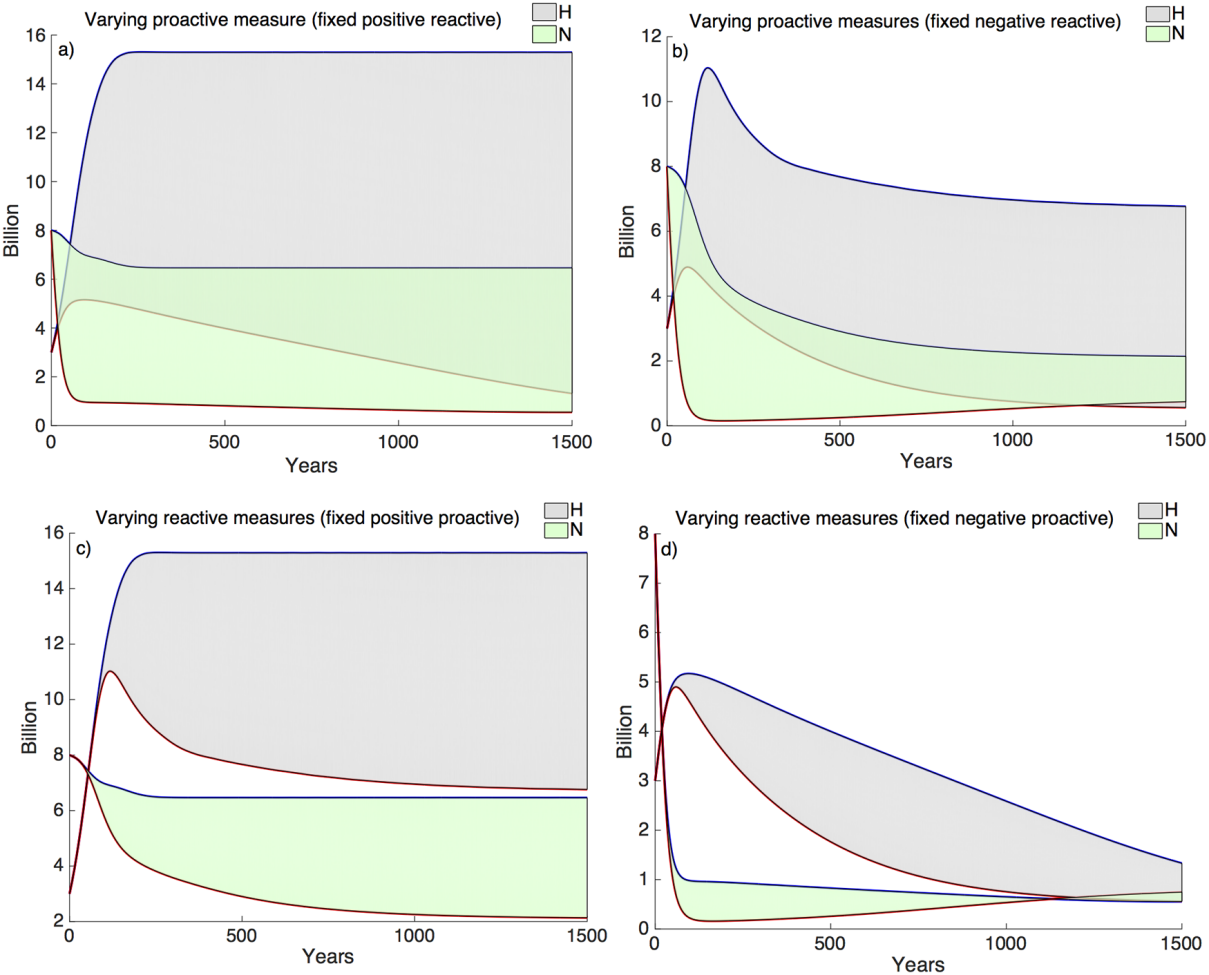
Proactive vs reactive approaches to sustainability. A proactive measure involves a decision that causes a change in either human population (*H*) or landscape dynamics (*N*, *A*), whereas a reactive measure responds to changes in the system. In this example, the rate of human-driven natural land degradation (*d*_*N*_) is proactive (subplots **a**, **b**) and the conservation restoration of *D* to *N* (*c_ND_*) is reactive (subplots **c, d**). The blue lines represent positive proactive decisions (subplots **a**,**b**) and positive reactive decision (subplots **c**, **d**). The red lines represent negative proactive decisions (subplots **a**, **b**) and negative reactive decisions (subplots **c**, **d**). The grey and green regions represent the range of outcomes for *H* and *N*, respectively. The proactive measure provides (or limits) options for the system, establishing a range of potential outcomes. If the decisions are obtuse with respect to future needs and ecosystem services (negative proactive decision), then reactive approaches cannot repair in later years (subplot **d**). However, in a changing environment, reactive measures are crucial to maintaining *N* and *H*. In subplot **b**, supply no longer meets demand and without conservation or restoration (low *c*_*ND*_), *H* collapses. Restoration efforts in a system with an initial positive proactive decision (subplot **c**) increase *N* and *H* for all scenarios (high *c_ND_*, blue line and low *c_ND_*, red line). In summary the plots illustrate that sustainability benefits from foresight (positive proactive decisions) and the ability to respond to a changing system (positive reactive decisions). Positive proactive, set *d*_*N*_ = 0.0001; negative proactive, set *d*_*N*_ = 0.01. Positive reactive, set *c_ND_* = 0.002; negative reactive, set *c_ND_* = 0.0001.

Factors that significantly alter *N*, and therefore the system at large, such as human-driven natural land degradation (*d*_*N*_) and conservation of natural land (*c_ND_*) play a critical role in the sustainability of the system. These two parameters also provide a comparison between proactive and reactive approaches for maintaining *N*. Here, a change in the rate of human-driven natural land degradation (*d*_*N*_) is a proactive approach, as it either prevents or increases degradation of land—the initial decision that alters the outcome of land area or human demography. The potential outcomes vary drastically over the range of *d*_*N*_ (Fig 4a). By contrast, land conservation (*c_ND_*) by conversion from degraded or unproductive land (*D*) to natural/‘semi-natural’ land (*N*) is a reactive approach that increases *N*, *A* and *H*, but requires a decrease in *N* beyond the limits of necessity to stimulate a conservation response—the secondary decision in response to the deterioration of land or human well-being.

When comparing proactive and reactive approaches, it is clear that both are necessary for the maintenance of ecosystem services (ES) and human well-being (Fig 4). Proactive measures determine whether future populations have options, controlling the opportunities for success or failure. Reactive measures work to either enhance or maintain the desired system. Fig 4, subplot c), shows potential human population and natural land outcomes over a range of reactive measures (*c_ND_*) with an initial positive proactive measure (low *d*_*N*_), whereas subplot d) shows the influence of reactive measures when the initial decision is negative (high *d*_*N*_). The area of natural land systems and the potential human population size is much greater when the proactive decision is positive, but in order to be sustained there must also be positive reactive decisions. Reactive measures are critical when the system is changing, particularly when there is a rapid decline in the system, which is illustrated in the comparison between subplots a) and b) in Fig 4. Without reactive measures the system will collapse (Fig 4b).

The current system experiences the greatest changes from reactive measures, primarily as a result of land conversion from productive natural/‘semi-natural’ land (*N*) to intensive agricultural land (*A*) (Fig C in S1 Text). The conversion of *N* to *A* is a reaction to increased human population size. Moreover, agricultural area allows the human population (*H*) to rapidly expand, involving a positive feedback between *H* and *A*. The positive feedback is interrupted when a negative feedback from *N* and insufficient ES is triggered. By contrast, reactive measures can play a role in stabilizing the system, as is the case with the restoration of natural land (*c*_*ND*_, Fig. C in S1 Text). Showing that reactive measures are most critical when the system is experiencing large transitions. However, in such cases there is the potential to overcompensate or act myopically, which may result in counteractive consequences.

#### Key factors

Of the seven parameters that have the greatest impact on the system and increase the likelihood of sustainability—a low biodiversity and ES threshold (*N*_*th*_); highly efficient use of agricultural land area (*E*_*A*_); a weak influence of natural and agricultural resources on the mortality rate (*q*); a moderate rate of conversion/conservation from degraded or unproductive land to natural land (*c*_*ND*_); a low rate of human-driven natural land degradation (*d*_*N*_); a lower demand for natural and agricultural resources (*η*_*N*_, *η*_*A*_)—five of them are directly related to human actions. *q* and *N*_*th*_ are assumed to be intrinsic feedbacks that alter human well-being through the quality and availability of resources; thus these parameters are not directly or indirectly related to human activities.

Despite the large difference in land cover and population levels in the long-term over the range of each of these key parameters, it is not evident until the end of the century that parameter selection (e.g.,value of *η*_*N*_ or *d*_*N*_) has contrastive outcomes. As can be seen from Figs 3 and 4, even in the worst case scenarios the human population follows the same trajectory for the first 50 years, only changing once there has been a significant decline in natural and ‘semi-natural’ area.

## Discussion

In an attempt to find drivers of population change and measures of sustainability, our model demonstrates that insufficient productive natural and ‘semi-natural’ land could be responsible for future declines in human population. In many cases, the system follows the same initial trajectory over a range of parameter values, making it difficult for individuals to assess, without a model, that one choice is preferable to another.

We suggest that ignoring the dependence of humans on natural ecosystem services would give an incomplete picture of human population and land dynamics. Likewise, human population dynamics have an unparalleled impact on the degradation of natural land. Although this might seem trivial, many individuals treat population growth as an exogenous factor when modelling ecosystem changes [39]. We show that the most efficient way to reduce negative human impacts on the system is to decrease the demand for resources, especially as the number of individuals increases over the next 80 years. This sentiment is shared by many others [40, 3, 41, 17, 42]. Ultimately, the well-being of the human population and the sustainability of ecosystem services is governed to a large extent by the rate of natural land decline, for which the interactions between human population size and natural land often comes to a pivotal point at approximately 38% of the Earth’s total ice-free surface area.

Interestingly, this tipping point corresponds with the estimated *N* requirements on a regional scale to maintain agricultural production [43]. The rapid degradation of productive natural/‘semi-natural’ land and resource consumption beyond the limits of exploitation cause an increase in mortality and the human population size declines. In such scenarios, agricultural yield is limited by a loss of ecosystem services and natural resources are over-exploited. The availability of resources does not meet the demands.

The decline in the human population predicted by our model follows a similar trend to that documented following the collapse on Easter Island [6, 30], where the population grew as resources were abundant until the land was degraded beyond its capacity, creating a population overshoot and decline. What makes this research particularly novel is the ability to combine feedbacks and concepts of population growth and land-use change into a simple dynamical model that fits well to data and can be used to evaluate alternative scenarios, explore potential collapses and offer ideas for societal and ecological sustainability.

In addition, the model shares many outcomes with the *Limit to Growth* (*LtG*) model first published in 1972 by Meadows et al. [44] and revisited by Turner in 2012 [45]. The *LtG* model predicted that the population should peak at a size 50% greater than in 2012 and 50 years later the population would collapse to half the size, in the comprehensive technology scenario. The collapse was attributed to a decline in non-renewable resources, which increased both the birth rate and the death rate. The increase in death rate that was presumed to drive the population to collapse in the *LtG* model, which is supported by our model. We also found a 50% increase in population from present to peak, yet our peak population occurs much later, at the beginning of the next century, and declines a minor 4% in the following 50 years. It seems unrealistic to use these models to accurately predict when the population will peak or collapse. The *LtG* model recognizes that the time scale is inaccurate and the UN projections shift with every new publication. However, valuable insight can be gained as to the cause of population change and the possibility of collapse.

The population estimate in the business as usual scenario of our model fits within the range of projections by the UN [46]. However, the decline in human population is caused by an increase in mortality, as a result of malnutrition. This contrasts the projections suggesting that the population decline will occur as a result of decreasing birth rates, as countries become more developed [46]. Our model shows a marginal decrease in the recruitment rate, but it is the lack of adequate resources that causes the population to level off and decline at the beginning of the next century.

An abundance of literature points to the need for human population growth and land degradation to be contained to secure a sustainable future [47, 11, 48]; we suggest natural land rather than agriculture is the key to sustainability. The increase in agricultural land in the model output, is consistent with projections and estimates of agriculture required to meet a growing population’s demands from the FAO, which projects a 70% increase from 2005 to 2050 [49]. Yet, it is interesting to note that increasing agriculture, without adequate natural land, does not benefit people in the long-term. When applied to real-world scenarios, this trend is exaggerated by the fact that most productive land is already used for agriculture. Therefore, further agricultural expansion would occur in marginal lands, which are more vulnerable to degradation and less productive.

When we consider the three categories of land cover modelled here: productive natural and ‘semi-natural’ land (i.e., forests, subsistence farms, grasslands), intensive agricultural land (i.e., permanent crops, arable land), and unproductive/degraded land (i.e., deserts, urban areas, over-exploited croplands), the conversion of natural land to agricultural land turns out to be counteractive to sustaining populations in the long run. Without adequate natural land to supply ecosystem services, the agricultural land yield and quality is too poor to provide any benefits to the population. Moreover, our projections are limited to the quantification of land area, which does not include shortages in resources or changes in demand or diversity in natural landscapes and ecosystem service benefits. Therefore, the model may underestimate the impact of ecosystem services on humans.

That is not to say that food production is not critical, we need food to maintain the population. However, this work, along with a growing body of literature, raises issue with the current view of large-scale monoculturalism and suggests a refocusing of food production, one that aims to increase yields and environmental integrity [17]. It is well-documented that agricultural land with adjacent natural area for the conservation of pollinators improves yields/incomes [50, 11] and small diversified farms produce more per area and are more secure than large monocultures [51, 52]. Suggesting that subsistence farming, for which the majority of the world’s population lives off [12], provides a productive ‘semi-natural’ land management alternative to intensive farming.

When it comes to biases in land valuation, there is a general consensus that agricultural land is both sociologically and economically more valuable. Despite there being a current excess of agricultural land [17], not considering the unequal distribution of food and resources, there is the tendency to believe that there is a need for further agriculture development and expansion [53]. However through model simulations, we find it is better to have a low perceived value of agriculture compared to a high perceived value of natural and ‘semi-natural’ land. This is due in large part to the ability to more rapidly convert land to agriculture than natural land. Therefore, a high perceived value of agriculture has an amplifying effect. Increasing agricultural land masks the need for natural ecosystems, providing high-yield food production that is able to feed a large population, and as such it is understandable that there is a bias for agricultural land. However, without supporting and regulating services, the yield from agricultural areas cannot be maintained.

Natural land degradation has a much greater impact than we as a society may recognize. Natural and semi-natural land provide water, timber, grazing lands, energy. Furthermore, there are numerous ecosystem services—such as water and air purification, soil stability, and waste recycling—that help regulate human well-being and sustain agriculture. Without natural and ‘semi-natural’ land and the accompanying services, agricultural land cannot be maintained, which creates a double loss. This may be surprising to many, but it is not unreasonable. Intensive agriculture makes up a small proportion of current land use (12%), but it is the prevailing solution for feeding the growing population. It should be noted that we assume technological improvements are static, which places a greater emphasis on natural land, something that has been discussed in a recent paper by Motesharrei and colleagues [3]. If technological innovations were to perpetually continue, then the human population would not have as great a need for natural land. Innovation played an immense role in population growth over the last half century, where agricultural area increased only 9%, but the yield increased 145% [31]. Yet, yield is stagnating throughout many high-income countries and we cannot rely solely on increasing agricultural production to sustain the population.

Additionally, our model includes societal factors that may impede or encourage sustainability. From a social and economic stand point, an argument can be made that sustainable management should implement an integrated approach of foresight and adaptability. As proactive approaches offer options in the future, either instituting a desirable or unfavourable future. However, a desirable proactive approach may not be sufficient. In a system that experiences drastic change (e.g., exponential population growth), reactive measures are required. The decisions, values or practices that were set in place decades or centuries ago may not meet the demands of the present or future.

We have made assumptions in this model that are contentious, specifically related to the link between recruitment and resource availability (Eq 4), using correlations to support our model. We make note that the existence of a correlation does not imply a direct mechanism, but we have used these correlations as possible indicators and have compared the assumptions over a range of historical and contemporary data. There are many factors that are related to one another, which makes a direct cause of human population change illusive. Many of these factors are themselves correlated and if consumption is not a direct factor in the demographic transition, than it acts as a proxy. Other models have used consumption patterns in various ways for modelling population growth, either directly through resource availability [30, 54] or in terms of inequality [8], suggesting that there is a strong bidirectional link between humans and the environment.

Applying a model with feedbacks between the human and Earth systems allows us to use information from the past and expand on it through the foresight of projection. The model can show us allows us to recognize when resources are being depleted beyond the ability to replenish, and offers a way of testing an array of changes to system that would not be possible otherwise.

## Supporting information

S1 Text Supplementary figures, table and parameterization. Description of parameter values, in addition to sources and data calibration. Further analyses are provided with respect to model simplification, model robustness and stability.
(PDF)
