# Supplementary material for: How ecological feedbacks between human population and land cover influence sustainability
Source: PLoS Comput Biol. 2018 Aug 17;14(8):e1006389. doi: 10.1371/journal.pcbi.1006389 (PMC6114924; doi:10.1371/journal.pcbi.1006389)
Supplement: S1 Text — Description of parameter values, in addition to sources and data calibration. Further analyses are provided with respect to model simplification, model robustness and stability. (PDF) [file pcbi.1006389.s001.pdf]

S1 Text: Supporting information

The MATLAB code and data used for the model simulations and figures are provided on Open Science Framework (OSF) and can be accessed through `https://osf.io/t3f8a/?view_only=e0b0f2826d1c4f7f8c40cdb789628bc6`.

Parameterization

The model was parameterized with human demographic data and land cover change data. Table A gives the parameters used under the business as usual scenario (Fig. 2 in the main text) and the range of parameters for potential scenarios (Fig. 3, 4 in the main text), including various degrees of conservation efforts, degradation rates, resource use, etc.

Human demography

The model was calibrated using World Bank population data for 1960, 1990, 1999, 2007 and 2014 [2]. The b and m were estimated to reflect recruitment rates and death rates in the indicated years, with the incorporated feedbacks from natural land (N) and agricultural land (A). Over the years population projections have varied widely, with an estimated peak population of one to 1 000 billion individuals [3]. However, in recent years, the population projections range has narrowed to between 9.4–10 billion in 2050 and 9.5–13.3B in 2100 [4]; the UN updated the population projection, estimating that the human population will continue to grow into the next century, reaching 11.2B in 2100 [4]. The simulated output for human population levels under baseline conditions are within 0.17B (less than three percent difference) of the empirical data.

The influence of N and A on the recruitment rate are estimated from evidence that malnourished populations have the highest birth rates [2], while also considering that famine limits fertility [5]; therefore, q_N and q_A should be chosen such that when A and N are very low, the recruitment rate decreases. Likewise mortality rates are impacted by the availability of resources. Prior to the 1900s, it would have been reasonable to assume that there was a causal link between resource availability and population growth. The rationale being that the population grows to a carrying capacity and then declines with insufficient resources, following a 'positive check' in Malthusian terms [6].

Additionally, it makes intuitive sense that with more land there is a greater need for labour to increase productivity [7, 8]. However, as technology and innovation becomes more integrated into the society, there is a shift in fertility, not just as a result of reduced labour needs, but also from a shift in lifestyle ideals. For example, farmers in the United States reduced their fertility rates, despite having abundant land, in order to provide a better quality of life for their children. It is due to this shift in lifestyles that we included the $\beta\eta_N$ term to reflect changes in social status and a shift in perception. The transition in the desire for offspring occurs as children become an expense, rather than an asset, with increases in wealth, education and consumption. Here we use resource demand (η_N) as a proxy for wealth, education and development.

Land cover dynamics

There is no consensus on land area composition. The land cover area was modelled to fit the various estimates of natural and 'semi-natural' land area (N), permanent crop and arable land area (A), and unproductive or degraded land area (D). The majority of data

Parameter	Description	Range	BAU Value
b	recruitment rate	0–0.05	0.025 (ind/yr)
β	demographic transition coefficient	0–0.05	0.025
q_N	labour coefficient for N	0–2	0.5
q_A	labour coefficient for A	0–2	0.75
m	adult mortality rate	0.001–0.05	0.0092 (ind/yr)
q	influence of resources (A and N) on mortality	0.03–0.42	0.11
E_N	efficiency of N use coefficient	0–2.5	0.75
E_A	efficiency of A use coefficient	0–2.5	0.87
η_N	demand for N resources	0–5	1.2 (ha/ind)
η_A	demand for A resources	0–2	0.22 (ha/ind)
N_{th}	biodiversity & ES threshold on productivity and well-being coefficient	0–13	5.3 (ha)
ϕ	N_b influence on A efficiency coefficient	0–3	0.49
p_N	collective perceived value of N	0–1	0.8
p_A	collective perceived value of A	0–1	0.5
$c_{_{NA}}$	conversion rate from A to N	0–0.001	0.00016 (ha/yr)
$c_{_{AN}}$	conversion rate from N to A	0–0.1	0.006 (ha/yr)
$c_{_{ND}}$	conversion rate from D to N	0–0.01	0.0007 (ha/yr)
$c_{_{AD}}$	conversion rate from D to A	0–0.01	0.0005 (ha/yr)
d_N	human-driven degradation rate, N	0–0.01	0.00045 (ha/yr)
d_A	over-exploitation rate, A	0–0.1	0.00065 (ha/yr)
r	N regeneration rate	0–0.001	0.0004 (ha/yr)
δ_N	nature-driven degradation rate, N	0-0.001	0.0005 (ha/yr)
δ_A	nature-driven degradation rate, A	0-0.001	0.0009 (ha/yr)
L	total land area	———	13 (ha)

Table A. List of model parameters, including the range of parameter values and parameter values for the business as usual (BAU) scenario. Data sources include FAOSTAT [1] and the World Bank Database [2], or are otherwise estimated in the below sections.

concerning land cover over the last 60 years comes from the FAO [1]; the FAO provides arable land/permanent cropland area (A); forest, meadows and pasture land area (N); and urban area (portion of D). Estimates for productive natural/'semi-natural' land (N) range from 6 to 8 billion hectares (Bha) in 2000 [9]. Between 1995 and 1999, A was mapped over approximately 1.7 to 1.8 Bha [10]. The area of unproductive/degraded land (D) holds the greatest uncertainty, with estimates for D ranging from 1 to 6 Bha globally [2, 11]. In 2007 the earth constituted 12.8% cropland, 53.7% 'natural' land and 31.5% unproductive/degraded land. D can be extrapolated from N and A, along with data describing crop abandonment, urbanization, deforestation, etc. The observed data points and projections are included in figure 2 from the main text.

Natural land (N)

Parameters were estimated using empirical data or calibrated to fit N decline over the past 50 years. For example, the time to recover an abandoned cropland (which falls under unproductive land) to natural grassland in terms of nutrient and soil organic matter recovery is 50 years [12]; therefore, $c_{AD} < 0.02$, with respect to biological limitations. Alternatively, the degradation from natural land to unproductive land decreases forest area by 0.2 Mha per year, therefore we assume $d_N NH > 0.0002$. Agricultural expansion has the greatest impact on natural land area. Each year 9.8 Mha of land is converted from forest to agriculture, therefore $c_{AN} NH \geq 0.0098$.

Demand for natural resources and agricultural resources (η_N and η_N, respectively) is based off of work from Wackernagel and colleagues, in addition to reports from the FAO on global consumption patterns [1, 13], showing hectares of area needed for resource demand per capita. Based on current calculations, in terms of biocapacity and population size, the actual demand for natural resources (η_N) is approximately six times that of agricultural demand (η_A).

There is much debate about the existence of a biodiversity or ecological threshold, but there are multiple papers suggesting a threshold at around 30% could result in extinction of species or a shift to an alternative stable state [14, 15]. Alternatively, Fahrig [16] shows that depending on a range of factors including fragmentation, movement, and growth—the extinction threshold can range from 1 to 99%. Therefore, we assume reduced ability to supply agriculture with required ecosystem services at $N \simeq 0.4L$, set $N_{th} = 5.3$. Since the data supporting a definitive threshold for which insufficient ecosystem services and biodiversity negatively impact land productivity and human well-being is inconclusive, we run model simulations over a range of N_{th} from 0 to 13 billion hectares to verify the robustness of the model. Figure A shows the equilibrium values for human population (H^*) and productive natural and 'semi-natural' land (N^*), with the resulting simulated natural land tipping point — the area of productive natural and 'semi-natural' land (N) in the model simulations that precedes a decrease in human population levels (H).

Intensive Agricultural Land (A)

The FAO has a wide array of statistics for agricultural land [1], including arable land area, permanent crop area, pasture area, consumption of agricultural products and per capita demand for agricultural land. It is estimated that 80% of deforestation is due to agriculture [17]; therefore, we assume the majority of conversion to agriculture land comes from natural land rather than degraded land, therefore $c_{AN} \gg c_{AD}$. By contrast, the conversion of agricultural land to natural land is insubstantial and as such $c_{NA} < c_{AD} \ll c_{AN}$.

The majority of urban land development occurs on A, at a rate of 1.6 to 3.3 Mha/yr [9], therefore we expect that $d_A(N)HA$ should be in the range of 0.0016–0.0033.

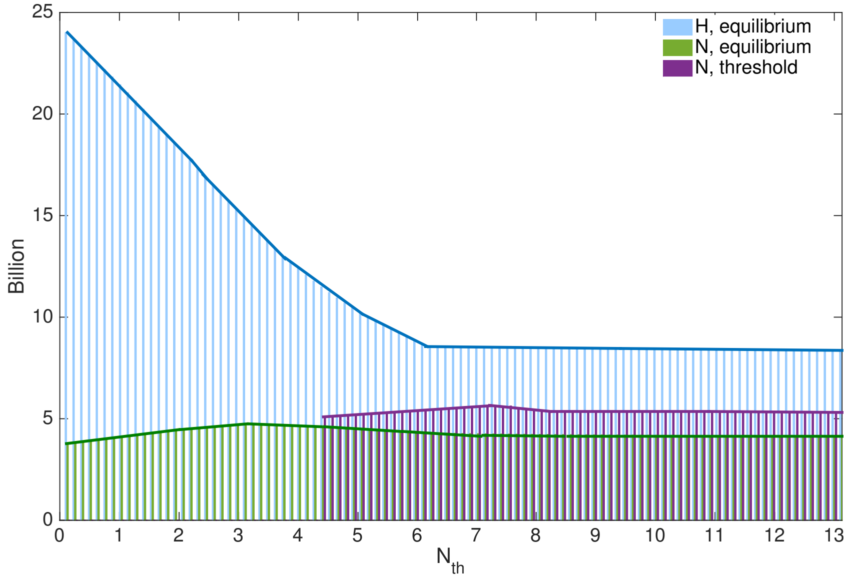

Fig A. The result showing human population (H) declines for $N < 5B$ may seem trivial considering the threshold for agricultural productivity (N_{th}) is set at 5.3; however, here we show that the equilibrium value of N is independent of N_{th} and that the threshold for productive natural and 'semi-natural' land is approximately 5B ha for N_{th} ranging from 4.2 to 13. This plot also demonstrates the strong association between human population (H) and N_{th}. Although, we cannot be certain of the human-well being and agriculture productivity response to surrounding natural resources, it is clear that how individuals use and acquire resources is critical to human well-being.

In addition, another 4 Mha of land is abandoned each year. Despite the continuous degradation of agricultural land, the area of permanent crops has not changed to a large extent in recent years, which suggests that the agricultural land that is abandoned or unproductive land is replaced by converting natural land into agriculture and that crop productivity plays an important role in feeding the population.

Unproductive/Degraded Land (D)

The work of Gibbs and Salmon [11] attempts to map global degraded land and it is estimated to be between 1 and 6 billion hectares. There is no general consensus on the area of degraded or unproductive land, however Lambin and colleagues project an increase of 30 to 87 Mha of D between 2000 and 2030 [9], which is consistent in our model (Fig. 2 in the main text). Furthermore, drought and desertification cause 12 Mha/yr to be lost every year [18], therefore in our simulations $dD/dt > 0.012$.

Simplifying the model

Figure B shows that η_N, m, N_{th}, b, q, E_N, η_A, $c_{_{ND}}$, E_A, d_N and p_A have the largest impact on the model system. It can also be seen that the model can be simplified by removing $c_{_{NA}}$ (unlikely and a slower rate of change); $c_{_{AD}}$ (unlikely and a slower rate of change); β, q_N and q_A (not strong enough to modify recruitment rate); r, δ_A, δ_N (humans have greater influence, slower rate of change).

Human practices move on a faster timescale than many natural processes, excluding stochastic natural disasters (not modelled here). Humans are thus controlling the

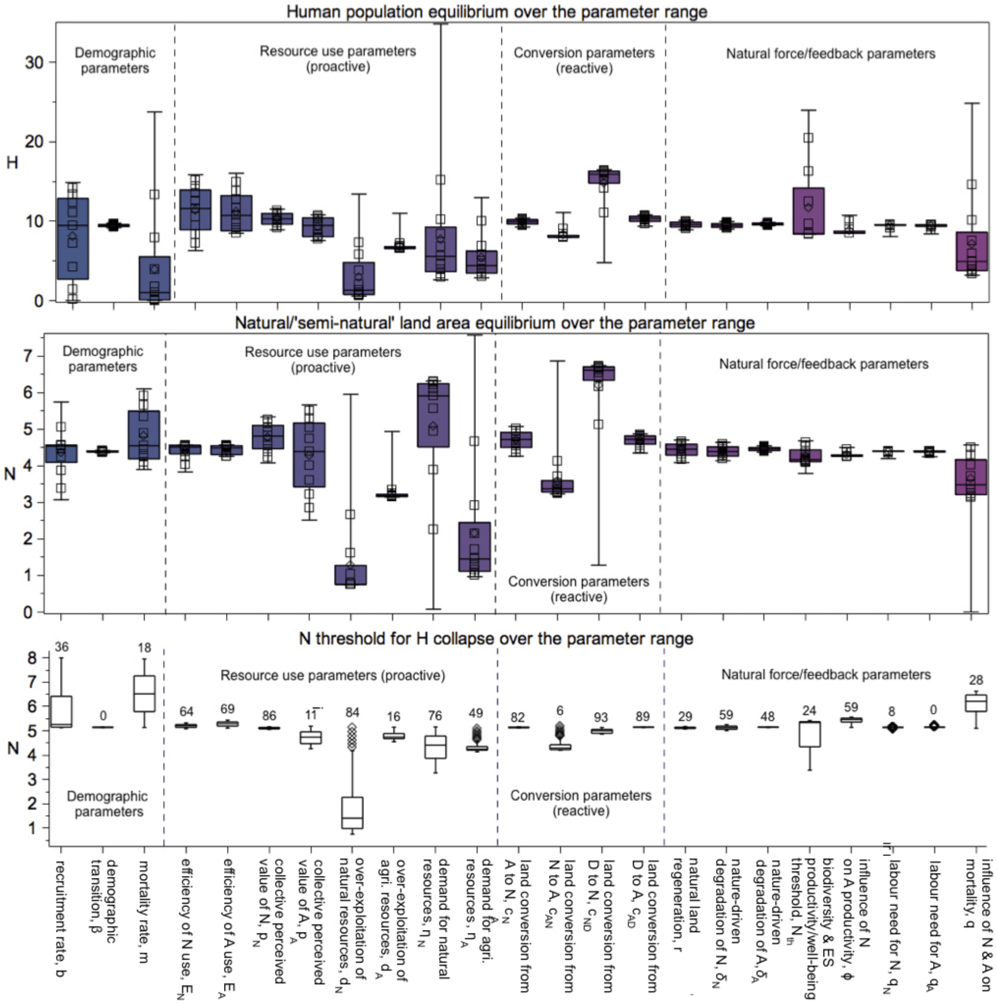

Fig B. Parameter analysis and model simplification. Parameters were varied one at a time, holding the other parameters constant, to acquire H^* and N^* from simulations after 1500 years. Box plot statistics were then applied to the simulation output. The top panel gives the human population (H) equilibrium for all possible parameter ranges. The middle panel gives the natural/'semi-natural' land area (N) equilibrium for all possible parameters. Finally, the bottom panel shows the N tipping point, for which there is a decline of at least one billion people after the threshold area of N is crossed. The number over the box plots indicates the percentage of parameter values where there is no decline. The resource use parameters (proactive) have the greatest overall impact on human population levels and land area, followed by demographic parameters, conversion parameters (reactive) and natural forces and feedbacks. Notwithstanding, the parameters that govern the biodiversity and ecosystem service threshold (N_{th}) and the influence of A & N on mortality (q) significantly impact human well-being.

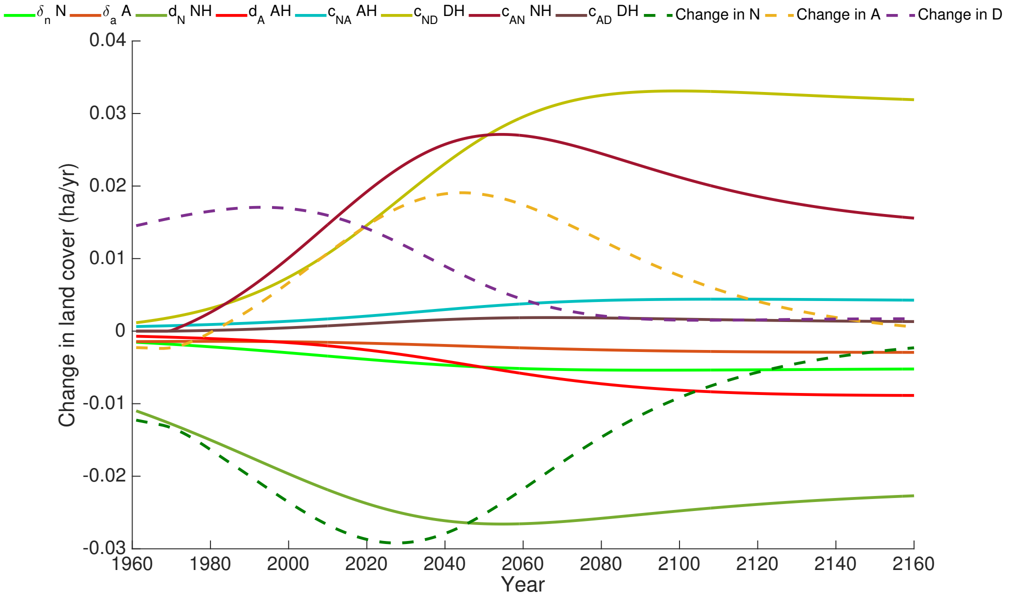

Fig C. Drivers of land change in the business as usual scenario. Initially, the change in land cover is governed by human-driven degradation of natural land (d_N, green solid line). However, as the demand for resources increases, conversion of natural land (N) to agricultural land (A) (c_{AN}, burgundy line), drives the decrease in N and increases A. The combined degradation of natural land from d_N and c_{AN}, induces conservation/restoration efforts (c_{ND}, olive green line). Finally, the land cover stabilizes with high conservation efforts, reduced demand for agricultural land and natural resources, as the population stabilizes. All other processes are negligible compared to d_N, c_{AN} and c_{ND}.

system, which explains why we do not see limit cycles. The driving factors, over the next two centuries, of intensive agricultural land (A) and productive natural and 'semi-natural' land (N) are given in figure C. Furthermore, from this plot it is clear that human population size (H) is driving the land cover changes. The model could essentially be simplified to a single variable, H. Natural land (N) would then act as a carrying capacity at approximately 5 billion hectares, depending on per capita land requirements and efficiency of use.

Stability Analysis

The model has multiple potential equilibrium values, which are highly-dependent on each other. The analytic solutions from this model are complex and do not yield intuitive results, therefore numeric simulations are more appropriate for this work. For the purpose of analysis we simplified the model to three variables, employing $D = L - N - A$.

There are two H equilibrium values, the trivial ($H^* = 0$) and the non-trivial:

$$H^* = \frac{E_A q A + E_N q N - L\beta\eta_n - N\eta_A q + L\ln\left(-\frac{b(\exp(-q_N N - q_A A - 1)}{m})\right)}{\eta_N q} \tag{S.1}$$

There are four possible equilibrium values for N, obtained when $dN/dt = 0$. In the

trivial case,

$$N^* = \frac{rL}{\delta_N + r} \tag{S.2}$$

When $H^* = 0$, $A^* = 0$, as there is no input into agricultural and a continuous loss to natural degradation. Otherwise, there are two viable equilibrium values, obtained by setting $dA/dt = 0$.

The stability of the equilibrium values are determined using a Jacobian matrix. However, the Jacobian matrix — given by taking partial derivative of each variable with respect to H, N and A — is too complex to give meaningful analytic solutions.

Limit Cycles

Given the complexity of the Jacobian matrix, it is difficult to obtain the eigenvalues analytically. The Muldowney criterion can be applied to determine under what conditions limit cycles might be generated [19]. The Muldowney criterion is an extension of the Bendixson criterion, which is used to preclude the presence of limit cycles in the model. Limit cycles exist for:

$$sup\left(\frac{\partial H}{\partial H}+\frac{\partial N}{\partial N}+\left|\frac{\partial A}{\partial H}\right|+\left|\frac{\partial A}{\partial N}\right|, \frac{\partial H}{\partial H}+\frac{\partial A}{\partial A}+\left|\frac{\partial N}{\partial H}\right|+\left|\frac{\partial N}{\partial A}\right|, \frac{\partial N}{\partial N}+\frac{\partial A}{\partial A}+\left|\frac{\partial H}{\partial N}\right|+\left|\frac{\partial H}{\partial A}\right|\right) < 0 \tag{S.3}$$

Based on the range of parameters, there are no parameter combinations which could potentially yield limit cycles. The rate of change for anthropogenic driven processes is much faster than that of ecological change. It is therefore possible to suggest that the system is driven by human consumption and demographics.

References

1. Food and Agriculture Organization of the United Nations. FAOSTAT. Food and Agriculture Organization of the United Nations; 2017.

2. The World Bank. World Bank Open Data, 2017; data retrieved from `https://data.worldbank.org`;.

3. Cohen JE. Population growth and earth's human carrying capacity. Science. 1995;269(5222):341.

4. United Nations, Department of Economic and Social Affairs. World Population Prospects: The 2015 Revision, Key Findings and Advance Tables. United Nations, Department of Economic and Social Affairs; 2015.

5. Gupta MD, Shuzhuo L. Gender Bias in China, South Korea and India, 1920-90: Effects of War, Famine and Fertility Decline. vol. 2140. World Bank Publications; 1999.

6. Malthus TR. An essay on the principle of population: or, A view of its past and present effects on human happiness. Reeves & Turner; 1888.

7. Bentley GR, Goldberg T, Jasieńska Gy. The fertility of agricultural and non-agricultural traditional societies. Popul Stud. 1993;47(2):269–281.

8. Easterlin RA. The conflict between aspirations and resources. Popul Dev Rev. 1976; p. 417–425.

9. Lambin EF, Meyfroidt P. Global land use change, economic globalization, and the looming land scarcity. Proc Natl Acad Sci USA. 2011;108(9):3465–3472.
10. Ramankutty N, Evan AT, Monfreda C, Foley JA. Farming the planet: 1. Geographic distribution of global agricultural lands in the year 2000. Global Biogeochem Cy. 2008;22(1).
11. Gibbs H, Salmon J. Mapping the world's degraded lands. Appl Geogr. 2015;57:12–21.
12. Burke IC, Lauenroth WK, Coffin DP. Soil organic matter recovery in semiarid grasslands: implications for the conservation reserve program. Ecol Appl. 1995;5(3):793–801.
13. Wackernagel M, Schulz NB, Deumling D, Linares AC, Jenkins M, Kapos V, et al. Tracking the ecological overshoot of the human economy. Proc Natl Acad Sci USA. 2002;99(14):9266–9271.
14. Huggett AJ. The concept and utility of 'ecological thresholds' in biodiversity conservation. Biol Conserv. 2005;124(3):301–310.
15. Estavillo C, Pardini R, da Rocha PLB. Forest loss and the biodiversity threshold: an evaluation considering species habitat requirements and the use of matrix habitats. PloS ONE. 2013;8(12):e82369.
16. Fahrig L. How much habitat is enough? Biol Conserv. 2001;100(1):65–74.
17. Tanentzap AJ, Lamb A, Walker S, Farmer A. Resolving conflicts between agriculture and the natural environment. PLoS Biol. 2015;13(9):e1002242.
18. United Nations Convention to Combat Desertification. Desertification: The invisible frontline. Secretariat of the United Nations Convention to Combat Desertification; 2014.
19. Li Y, Muldowney JS. On Bendixsons Criterion. J Differ Equ. 1993;106(1):27–39.
